# The Expression and Prognostic Value of Cancer Stem Cell Markers, NRF2, and Its Target Genes in TAE/TACE-Treated Hepatocellular Carcinoma

**DOI:** 10.3390/medicina58020212

**Published:** 2022-02-01

**Authors:** Duurenjargal Tseeleesuren, Hui-Hua Hsiao, Rajni Kant, Yu-Chuen Huang, Hung-Pin Tu, Chih-Chung Lai, Shiu-Feng Huang, Chia-Hung Yen

**Affiliations:** 1 Graduate Institute of Medicine, School of Medicine, Kaohsiung Medical University, Kaohsiung 80708, Taiwan; duurenjargal.ts@gmail.com; 2 Department of Hematology, School of Medicine, Mongolian National University of Medical Sciences, Ulaanbaatar 14210, Mongolia; 3 Natural Product Libraries and High-Throughput Screening Core Facility, Kaohsiung Medical University, Kaohsiung 80708, Taiwan; rajnihpmicro@gmail.com (R.K.); t139725846aa@yahoo.com.tw (C.-C.L.); 4 Division of Hematology and Oncology, Department of Internal Medicine, Kaohsiung Medical University Hospital, Kaohsiung 80708, Taiwan; huhuhs@kmu.edu.tw; 5 Center for Cancer Research, Kaohsiung Medical University, Kaohsiung 80708, Taiwan; 6 Faculty of Medicine, Kaohsiung Medical University, Kaohsiung 80708, Taiwan; 7 Cancer Center, Kaohsiung Medical University Hospital, Kaohsiung 80708, Taiwan; 8 School of Chinese Medicine, China Medical University, Taichung 404333, Taiwan; yuchuen@mail.cmu.edu.tw; 9 Department of Medical Research, China Medical University Hospital, Taichung 404332, Taiwan; 10 Department of Public Health and Environmental Medicine, School of Medicine, College of Medicine, Kaohsiung Medical University, Kaohsiung 80708, Taiwan; p915013@kmu.edu.tw; 11 Graduate Institute of Natural Products, College of Pharmacy, Kaohsiung Medical University, Kaohsiung 80708, Taiwan; 12 Institute of Molecular and Genomic Medicine, National Health Research Institutes, Miaoli 35053, Taiwan; sfhuang@nhri.edu.tw; 13 Doctoral Degree Program in Toxicology, College of Pharmacy, Kaohsiung Medical University, Kaohsiung 80708, Taiwan; 14 Drug Development and Value Creation Research Center, Kaohsiung Medical University, Kaohsiung 80708, Taiwan

**Keywords:** hepatocellular carcinoma, transcatheter arterial chemoembolization, NRF2, NQO1, CD133, prognostic marker

## Abstract

*Background and Objectives*: Activation of NRF2, a key transcription factor of cytoprotectant against oxidative stress, and its target genes are associated with aggressive tumor progression, metastasis and poor survival. In addition, NRF2 signaling mediates cancer stem cell (CSC)-like properties in hepatocellular carcinoma (HCC) cells. Moreover, CSCs have been associated with HCC onset and unfavorable prognosis. Transcatheter arterial embolization (TAE) and/or transcatheter arterial chemoembolization (TACE), which attempt to restrict blood supply to diminish tumor growth, can create a hypoxic environment. However, its effect on NRF2 signaling and CSC marker CD133 in the context of prognosis of HCCs have not been investigated. Therefore, we studied the possible role of the expressions of NRF2, its target genes and CSC markers CD133 and EpCAM on the survival of HCC patients after TAE/TACE. *Materials and Methods*: RT-qPCR was performed with 120 tumor (T) and adjacent tumor (N) tissue pairs. Expression of a single marker or combination was assessed for associations with survival of HCC patients after TAE/TACE. *Results*: The result of multivariate Cox regression showed that vascular invasion (HR, 1.821; *p* = 0.015), metastasis (HR, 2.033; *p* = 0.049) and CD133 overexpression (HR, 2.013; *p* = 0.006) were associated with poor survival. In a Kaplan–Meier survival analysis, patients with high expression of CD133 had shorter overall survival (OS) than those with low expression of CD133 in post-TAE/TACE HCC (*p* < 0.001). In contrast, neither NRF2 nor components of its signaling pathway correlated with survival. Combination marker analysis showed that co-expression of NQO1 and CD133 was associated with poor outcome. *Conclusions*: This study suggests that analyzing the expression status of CD133 alone and co-expression of NQO1 and CD133 may have additional value in predicting the outcome of TAE/TACE-treated HCC patients.

## 1. Introduction

Hepatocellular carcinoma (HCC) incidence has been escalating globally over the past two decades; it is the sixth most prevalent cancer and the third leading cause of cancer-related deaths. In 2018, the estimated number of HCC-related mortality accounted for more than 800,000, and it is predicted to increase by more than 1 million in the upcoming 20 years [1]. Various treatments, including targeted therapies and immunotherapies, have been shown to be available based on the stage of HCC [2]. Intermediate-stage HCCs have been treated with the widely accepted therapeutic option of transcatheter arterial embolization (TAE) or transcatheter arterial chemoembolization (TACE) as downstaging or bridging therapy prior to resection and liver transplantation [3]. The modality of these treatments results in ischemic necrosis due to arterial embolization. However, the overall survival after TAE/TACE is 11 to 45 months [4], and the recurrence rate of tumors after TAE/TACE is common, being as high as 45% within two years [5]. Moreover, the biological behavior of the tumors post-treatment is variable, and in some cases selecting TAE/TACE treatment exacerbates hypoxia and induces more aggressive tumors [6]. Therefore, a better understanding of tumor biology and markers that can better predict patient survival after TAE/TACE would significantly impact the management of the disease.

Nuclear factor E2-related factor 2 (NRF2) is a transcription factor known to be a key regulator of cytoprotectants against oxidative stress. Under physiological conditions, it is bound to Kelch-like ECH-associated protein 1 (KEAP1) in the cytosol; oxidative stress causes dissociation of NRF2 from KEAP1. As a result, NRF2 translocates into the nucleus, and activates transcription of downstream target genes. The activation of target genes provides cytoprotection against oxidative stress [7,8]. Several studies have shown that overexpression of NRF2 and its target genes in cancer cells might protect cancer cells from oxidative stress and apoptosis and result in cancer cell proliferation, survival, and resistance to therapy [9,10]. In 2016, Wang et al. determined that elevated expression of NRF2 positively correlated with HCC metastasis [11]. Previous studies found that HCC patients overexpressing the NRF2 target gene NAD(P)H: quinone oxidoreductase 1 (NQO1) had a significantly lower survival rate than those who did not [12], and that overexpression of this gene might act as a potential biomarker for early diagnosis and therapy [13]. In addition, a study demonstrated that the malignant phenotype of HCC is associated with higher expression of the NRF2 target gene glutamate–cysteine ligase catalytic subunit (GCLC), and that its level can predict clinical outcome after resection, even in early-stage HCC [14]. The effect of TAE/TACE is to induce a high degree of intertumoral hypoxia due to vascular occlusion, and hypoxic HCC cells are known to be resistant to chemotherapeutic agents [15]. Hypoxia has been shown to lead to activation of NRF2 [16]. A recent study showed that HCC with NRF2 mutation exhibited rapid local progression after TAE, and overexpression of NRF2 in the HCC cell line showed resistance to ischemia. Furthermore, inhibition of NRF2 resulted in a sensitizing effect of ischemia on the growth of the HCC cell line [4]. However, whether the expression of NRF2 and its target genes contribute to patient survival after TAE/TACE in HCC patients is still unknown.

Cancer stem cells (CSCs) function as a proliferation pool that appears to contribute greatly to tumor formation, recurrence and metastasis [17]. Previously, several cell surface markers such as CD133 and epithelial cell adhesion molecule (EpCAM) were identified as CSC markers in HCC [18]. Studies demonstrated that CSC markers might be associated with HCC onset, metastasis and unfavorable prognosis [19,20]. Earlier Zeng et al. showed that the expression of EpCAM and CD133 was significantly higher in post-TACE HCC tissues compared to adjacent tissues [6]. Nahm et al. found that the expression of CSC markers increased under TACE-induced hypoxia [21]. To date, the clinical value of CD133 in TAE/TACE-treated HCCs is insufficient, and no study has compared the correlation between CD133 and the prognostic value with respect to post-TAE/TACE HCCs. Moreover, Rhee et al. demonstrated that HCC-expressing EpCAM and CK19 frequently showed treatment resistance to transarterial chemoembolization and a worse outcome than carcinomas without chemoembolization [22]. In addition, NRF2 has been reported to regulate the expression of genes important for stemness in cancer cells to promote malignancy [23]. A recent study has suggested that the NRF2 signaling pathway regulates stem-like markers in sorafenib-resistant HCC cells [24].

Previous studies have shown that hypoxia induces the expression of NRF2, its target genes and CD133 in various cancers [25,26]. Interestingly, TAE/TACE has been shown to exacerbate hypoxia, which induces various factors leading to adverse clinical outcomes in TAE/TACE-treated HCC [27]. However, to our knowledge, no study has investigated the prognostic significance of CD133 in TAE/TACE-treated HCC patients. Moreover, the correlation of the expression of NRF2 and its target genes with prognosis in patients after TAE/TACE has not been reported. Therefore, the mRNA expression levels of the CSC markers CD133 and EpCAM, and NRF2 and its target genes NQO1, HO-1, GCLC and GCLM alone and in combination were analyzed to evaluate their association with the prognosis of TAE/TACE-treated HCC patients.

## 2. Materials and Methods

### 2.1. Ethical Approval

The study protocol was approved by the institutional review board of Kaohsiung Medical University Hospital and the Taiwan Liver Cancer Network user committee (TLCN, accessed on 14 January 2022), which was in compliance with the Declaration of Helsinki. Informed consent was obtained for sample and data collection from each patient or their legal representative.

### 2.2. Source of RNA Samples 

Total RNA of HCC tumor (T) and adjacent tumor-free liver (N) specimens were provided by the TLCN. These samples were collected from 120 patients previously treated with transcatheter arterial embolization (TAE) and/or transcatheter arterial chemoembolization (TACE). Of these patients, 24 received surgery, 3 alcohol injection, 5 radiofrequency ablation (RFA) and 1 medium chain triglycerides (MCT) before TAE/TACE. The clinical data associated with the specimen was also provided by the TLCN. Tumors were staged according to the 7th edition of the American Joint Committee on Cancer (AJCC). 

### 2.3. Reverse Transcription–Quantitative Real-Time PCR (RT-qPCR) Analysis

Reverse transcription (RT) was performed to obtain cDNA using the High-Capacity cDNA Reverse Transcription Kits (Applied Biosystem, Waltham, MA, USA) according to the manufacturer’s protocol. Real-time PCR was performed in the 7900HT Fast real-time PCR (Applied Biosystems) using the Fast SYBR^TM^ Green Master Mix protocol (Applied Biosystems by Thermo Fisher Scientific, Waltham, MA, USA). Quantitative values were obtained by the threshold cycle (Ct) value, which was normalized using the mean Ct value for the reference gene, TATA-box-binding protein (TBP). The ratio of expression of each target gene in the paired tumorous (T) and non-tumorous (N) tissues was calculated. A ratio of relative gene expression T/N of ≥1.5 was considered positive for high expression (overexpression) in the tumor compared to the corresponding tumor-adjacent tissue. The primer sequences used in this study are described in Table 1.

### 2.4. Statistical Analysis

All statistical analyses and graphs were performed using IBM^®^ SPSS^®^ Statistics software version 25.0 (SPSS Inc., Chicago, IL, USA). The statistical significance of the associations between the clinicopathological parameters and the expression of the markers was evaluated using the χ^2^ test and the Fisher exact test. Overall survival (OS) was defined as the time from the date of surgery to death or the date of last visit to the hospital. OS was constructed using the Kaplan–Meier method, and the log-rank test compared the resulting curves. Cox proportional hazard regression was used to assess independent predictors of OS rates in HCC patients. In addition, parameters that were significant in univariate analysis were examined by stepwise multivariate analysis. All statistical tests were two-sided, with the threshold for significance defined as *p* < 0.05. 

## 3. Results

### 3.1. Baseline Characteristics of Patients

The present study comprised 120 patients previously treated with TAE/TACE, who subsequently underwent surgical resection of HCCs. The demographic and clinical characteristics of the HCC patients after TAE/TACE are shown in Table 2. The median age of the study population was 58.0 years. A total of 55 out of 120 patients (45.8%) were older than 60 years, 97 (80.8%) were male, and 71.7% had serum AFP levels of less than 400 ng/mL. The mean tumor size was 5.8 cm (median of 4.15 cm), and 51 (42.5%) patients presented tumors larger than 5 cm. According to Edmondson–Steiner grading (ES), 79 (65.8%) patients had predominantly well and moderately differentiated HCCs. Of the study participants, 70 (58.3%) had a solitary tumor mass. Vascular invasion was noted in 74 (61.7%) cases. Based on the AJCC/UICC 7th edition staging system, 87 (72.5%) cases were late stage HCCs. Liver cirrhosis was present in 66 (55.0%) of all cases. The main disease etiology was hepatitis B virus (HBV) infection in 77 (64.2%) cases. Metastasis was present in 10 (8.3%) patients. The median follow-up time was 47.01 months (interquartile range (IQR), 15.08–108.68). 

### 3.2. Association of mRNA Expression with Clinicopathologic Characteristics

The mRNA expression levels of NRF2 and its target genes, as well as the CSC markers EpCAM and CD133, were measured in 120 matched pairs of HCCs (T)/adjacent tissue (N) samples by RT-qPCR. A relative gene expression ratio (T/N) at or above 1.5 was considered positive for overexpression in the tumor compared to the corresponding tumor-adjacent tissue. The results showed that overexpression of NRF2, NQO1, HO-1, GCLC and GCLM was detected in 32 (26.7%), 85 (70.8%), 18 (15%), 40 (33.3%) and 50 (41.7%) cases, respectively (Supplementary Table S1). Concurrently, the expression of CSC markers EpCAM and CD133 were detected in 48 (40.0%) and 25 (20.8%) cases, respectively. Next, we examined the potential association of NRF2 and its related genes, the CSC markers EpCAM and CD133 with following clinicopathological variables of HCC: gender, age, smoking, alcohol consumption, serum AFP level, tumor size, ES grade, number of tumors, vascular invasion, pathology stage, cirrhosis, viral status, and metastasis. The clinicopathological characteristics of the patients with altered expression of CSC markers are summarized in Table 3. The results on NRF2 and its target genes are summarized in Supplementary Table S2. High expression of EpCAM was observed in patients who were younger than sixty years of age (*p* = 0.001), under the cut-off value of serum AFP (*p* = 0.007), multiple tumors (*p* = 0.036), and was more common in HBV-infected HCC patients (*p* = 0.015). High expression of CD133 was associated with a serum AFP level below 400 ng/mL (*p* = 0.043) and cases with multiple tumors (*p* = 0.013). In addition, CD133 expression was significantly higher in HCC with vascular invasion (*p* = 0.039) and without cirrhosis (*p* = 0.013).

### 3.3. Expression of CSC Markers Associated with NQO1 Expression in Post-TAE/TACE HCCs

EpCAM was expressed in forty-one (85.4%) cases that showed high expression of NQO1. The association between EpCAM and NQO1 was statistically significant (*p* = 0.011). Twenty-two (88.0%) patients who had high expression of NQO1 also had high expression of CD133, which was statistically significant (*p* = 0.047). No other correlations were found between CSC markers with NRF2 and its other target genes (Table 4).

### 3.4. Expression of CD133 Is an Independent Prognostic Factor in HCC Patients after TAE/TACE 

**Figure 1:**
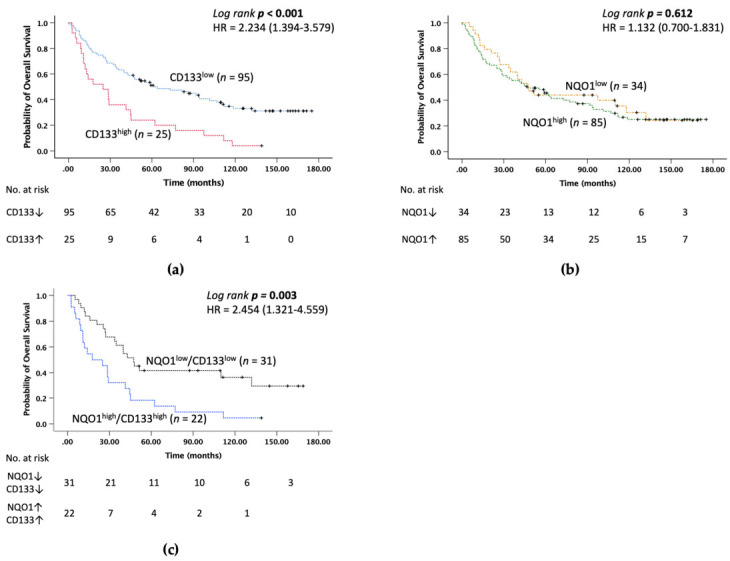
Survival analysis of CD133, NQO1 and co-expression of NQO1/CD133 using Kaplan–Meier method and *p* values determined by log-rank test. Association between overall survival and (**a**) CD133 expression, (**b**) NQO1 and (**c**) co-expression of NQO1 and CD133 in patients with post-TAE/TACE HCC.

The relationship between the expression of NRF2, its target genes, and CSC markers at the mRNA level and OS of HCC patients after TAE/TACE was further assessed by survival analysis. The result of the analysis showed that among all markers, patients with high expression of CD133 had significantly shorter OS than patients with low expression of CD133 (median survival 24.9 months versus 61.56 months) (*p* < 0.001, Figure 1a). Overexpression of NRF2, its target genes and the CSC marker EpCAM showed no significant impact on OS (data not shown).

Following the above findings, a univariate analysis was performed to correlate the expressions of the markers and clinicopathological parameters with OS of the 120 HCC patients after TAE/TACE. The results showed that an AFP level greater than 400 ng/mL (*p* = 0.011), tumor size greater than 5 cm (*p* = 0.004), late-stage HCC (*p* = 0.007), HCC with vascular invasion (*p* = 0.001), HCC with metastasis (*p* = 0.024) and high expression of CD133 (*p* = 0.001) were associated with decreased OS time. In addition, a multivariate survival analysis was conducted for all significant parameters found in the univariate survival analysis. The results showed that high expression of CD133 served as a significant independent prognostic factor for poor OS in HCC after TAE/TACE (HR, 2.013; 95% CI, 1.223–3.314; *p* = 0.006), as well as vascular invasion (HR, 1.821; 95% CI, 1.124–2.9.52; *p* = 0.015) and metastasis (HR, 2.033; 95% CI, 1.002–4.125, *p* = 0.049) (Table 5).

Due to the complex nature of cancer, a combination of markers have been shown to better predict survival. Therefore, we performed a univariate survival analysis of NRF2 and its target genes with combinations of CSC markers EpCAM and CD133 (e.g., NRF2/CD133 and NRF2/EpCAM, etc.) (data not shown). The survival analysis showed that overexpression of NQO1 alone had no significant association with OS (Figure 1b), but higher co-expression of NQO1/CD133 was associated with worse OS than low expression of NQO1/CD133 (Figure 1c). In addition, associations of high expression of CD133 with clinical outcomes which were significant in the univariate analysis were also investigated (Supplementary Table S3). Because of the limited number of patients in the subgroups, including HO-1/EpCAM, HO-1/CD133, GCLC/CD133 and metastasis/CD133, Cox proportional univariate analysis was performed only for subgroups with more than 10 patients. The result showed that the prognostic profile was associated with a significantly higher hazard ratio for patients with high expression of NQO1 with CD133 (HR, 2.454; 95% CI, 1.321–4.559; *p* = 0.004).

## 4. Discussion

TAE/TACE is a locoregional therapy for hepatocellular carcinoma that contributes to tumor shrinkage and downstaging or bridging before resection and liver transplantation in intermediate-stage HCCs [3]. After TAE/TACE, the outcome is different for each individual and challenging to predict, and recurrence is common. This study was conducted to determine a feasible role of post-TAE/TACE in HCC with the expression levels of NRF2, its target genes and CSC markers under an hypoxia microenvironment. To investigate this, the expression levels of NRF2, NQO1, HO-1, GCLC, GCLM, EpCAM and CD133 were analyzed in RNA samples from 120 pairs of HCC and adjacent tissues. To the best of our knowledge, this is the first study to report the correlation of NRF2, its target genes and CSC marker CD133 with expression characteristics and prognostic value in HCC patients after TAE/TACE. 

In recent years, cancer signaling pathways and CSC markers have gained importance as predictors of cancer prognosis and as targets for cancer prevention and treatment. Several studies have shown that high expression of CSC markers CD133 and EpCAM is associated with poor prognosis in HCC [20,28,29]. However, there are few reports on the correlation between the expression of CSC markers CD133 and EpCAM in TAE/TACE-treated HCC. Previous reports found that CD133 and EpCAM were highly expressed in TACE-treated HCC. Zeng et al. analyzed CSC markers in 16 TACE-treated and 23 untreated HCC by immunohistochemistry (IHC). The results showed that the expression of EpCAM and CD133 was significantly higher in the TACE group than in the group without TACE. It also showed that high EpCAM but not CD133 expression was associated with tumor recurrence [6]. Increased expression of CD133 and EpCAM was also observed by Nahm et al. using IHC in 10 patients with preoperative TACE compared to 36 patients without TACE [21]. In our study analyzing mRNA expression from 120 pairs of HCC and adjacent tissue samples, overexpression of CD133 was associated with tumor number, vascular invasion and cirrhosis. In addition, we found that CD133 overexpression was an independent prognostic marker that correlated with lower OS. The association between CD133 expression and prognosis in TAE/TACE-treated HCC has not been previously investigated. For EpCAM, the results showed that overexpression of EpCAM was significantly associated with age, number of tumors and viral status, but not with OS. Rhee et al. showed that HCC expressing CK19, EpCAM or CAIX had resistance to TACE with worse outcomes [22]. The difference in EpCAM expression in our study may be due to differences in study participants, data interpretation and methods used. In the aforementioned study, EpCAM expression in samples from Korean patients was determined by IHC and compared with/without the TACE group for outcome. Importantly, EpCAM expression was significantly associated with disease-free survival in their study, but not with OS. In addition, it has been postulated that the expression characteristics of stem cell markers in stem cell-specific carcinogenic cells may be diverse in each HCC. This could be due to the heterogeneity of activated signaling pathways of normal stem cells that these tumor-initiating cells come from [6]. Further studies with independent cohorts are needed to substantiate our findings. 

Previous meta-analyses have shown that overexpression of NRF2 is an unfavorable prognostic factor for several solid malignancies [30,31]. The association between NRF2 expression and poor prognosis has also been shown in HCC [9,32]; however, NRF2 association in residual tumor after TAE/TACE in HCC has not been reported so far. Only 1 study reported that the mutation in the NRF2 pathway leads to rapid tumor progression in HCC tumors treated with TAE/TACE. This suggests that the alteration of the NRF2 pathway contributes to resistance to ischemia [4]. We observed overexpression of NRF2 in 32 (26%) TAE/TACE-treated HCC patients. OS and other clinicopathological parameters were not significantly associated with NRF2 overexpression status. Furthermore, the NRF2 target genes NQO-1, HO-1, GCLC and GCLM also showed no significant association between expression level and clinicopathological features in TAE/TACE-treated HCC patients. Several studies reported that the expression of NRF2, NQO-1 and GCLC was associated with poor survival and clinicopathological features in HCC [12,13,14]. The expression of HO-1 remains controversial in HCC. Park et al. showed that expression of HO-1 was not associated with patient survival in human HCC [33]. In contrast, another study reported that expression of HO-1 was associated with favorable disease-free survival in HBV-HCC patients [34]. The association between GCLM expression and overall survival has not been reported in HCC. The different results of the expression of NRF2 and its target genes in our study might be affected by the changes in the hypoxic microenvironment of HCC triggered by TAE/TACE. Previous study used HCC patient samples, not TAE/TACE-treated HCC samples, to examine OS. No other study examined the expression correlation of NRF2 and its target genes in relation to TAE/TACE-treated HCC. The only study on NRF2 in TAE/TACE-treated HCC showed that NRF2 mutation leads to rapid tumor progression in 14% of patients, but this study also did not show OS data [4]. The microenvironment of an HCC tumor after treatment with TAE/TACE is vastly different from that of HCC without TAE/TACE. Therefore, this study is the first to analyze the prognostic role of NRF2 and its target genes. The results do not support the fact that these genes can be used as a prognostic factor in TAE/TACE-treated HCC. 

In recent years, it has been shown that a combination of markers may provide a better prognosis than a single marker [35,36]. In this study, we checked the different combinations of genes and their expression. Only the NRF2 target gene NQO1 showed a significant correlation in combination with CD133. However, NRF2 and other target genes did not show significant correlation with OS. Therefore, we hypothesize that the regulation of NQO1 in TAE/TACE-treated HCC should have a mechanism independent of NRF2; however, further studies are needed. The results suggest that targeting CD133 in combination with NQO1 may be a novel therapeutic strategy for intermediate-stage HCC with preoperative TAE/TACE. Additionally, Granito et al. demonstrated that elevation of transaminases after superselective cTACE can be used as a predicted treatment response to cTACE in HCC [37]. Our study showed that the overexpression of CSC marker CD133 alone and in combination with NQO1 correlated with poor OS of TAE/TACE-treated HCC. Therefore, it might be beneficial to determine the levels of ALT and AST, as well as the significant markers for prognosis of TAE/TACE-treated HCC as determined in our study.

Our study has limitations. The first is that all HCCs that participated in this study were pretreated with TAE/TACE and surgically resected. HCCs without TAE/TACE were not included, and may have resulted in selection bias. A comparative study of patients treated with and without TAE/TACE needs to be conducted to evaluate the further association of NRF2, its target genes, and CSC markers. Second, since all patients were of Asian descent (Taiwanese), similar studies are necessary in other continents or regions. Third, due to the small sample size, it was not possible to confirm whether a combination of NQO1/CD133 had a higher predictive power than the CD133 marker alone. Therefore, further research studies are needed to assert the above result fully. 

## 5. Conclusions

Our findings suggest that CD133 is an independent predictor of prognosis in patients treated with TAE/TACE HCC. We also demonstrated a significant association between expression levels of NQO1 and CD133. In addition, the co-expression of NQO1 and CD133 could be valuable markers for predicting the prognosis of patients with TAE/TACE-pretreated HCC. Thus, evaluating the expression of NQO1 and CD133 could provide useful information for clinicians to select appropriate treatment options for TAE/TACE-treated HCC patients. 

## Figures and Tables

**Table 1:** List of primer sequences used in this study.

Gene	Forward (5′→3′)	Reverse (5′→3′)
NRF2	AGACGGTATGCAACAGGACA	ACCATGGTAGTCTCAACCAGC
NQO1	TGCAGCGGCTTTGAAGAAGAAAGG	TCGGCAGGATACTGAAAGTTCGCA
HO-1	GCCAGCAACAAAGTGCAAG	GAGTGTAAGGACCCATCGGA
GCLC	CTGGGGAGTGATTTCTGCAT	AGGAGGGGGCTTAAATCTCA
GCLM	AGTGGGCACAGGTAAAACCA	CTCGTGCGCTTGAATGTCAG
EpCAM	CAGAACAATGATGGGCTTTATG	GCAGTCCGCAAACTTTTAC
CD133	TGGATGCAGAACTTGACAACGT	ATACCTGCTACGACAGTCGTGGT
TBP	CAGAAGTTGGGTTTTCCAGCTAA	ACATCACAGCTCCCCACCAT

**Table 2:** Baseline characteristics of the patients in the study.

Parameters	Value
Total cases, *n*	120
Age (year, mean, median)	
Mean ± SD Median, IQR	58.24 ± 12.09 58 (52–68)
Age (years)	
<60 ≥60	65 (54.2%) 55 (45.8%)
Gender, *n* (%)	
Male Female	97 (80.8%) 23 (19.2%)
Smoking, *n* (%) ^a^	
No Yes	59 (49.2%) 60 (50.0%)
Alcohol consumption, *n* (%) ^a^	
No Yes-low Yes-high	70 (58.3%) 18 (15.0%) 31 (25.8%)
AFP (ng/mL) ^b^	
Mean ± SD Median, IQR	9109.35 ± 50,338.77 34.01 (8.10–733.25)
AFP (ng/mL) ^b^	
<400 ≥400	86 (71.7%) 32 (26.7%)
Tumor size (cm)	
Mean ± SD Median, IQR	5.80 ± 4.29 4.15 (2.70–8.45)
Size, *n* (%)	
<5 cm ≥5 cm	69 (57.5%) 51 (42.5%)
Edmondson–Steiner (ES) grade, *n* (%)	
Well (I and II) Poor (III and IV)	79 (65.8%) 41 (34.2%)
Number of tumors, *n* (%)	
Solitary Multiple	70 (58.3%) 50 (41.7%)
Vascular invasion, *n* (%)	
Absent Vascular Invasion	46 (38.3%) 74 (61.7%)
Pathology stage, *n* (%)	
Early (I) Late (II, III and IV)	33 (27.5%) 87 (72.5%)
Cirrhosis, *n* (%)	
No Yes	54 (45.0%) 66 (55.0%)
Viral status, *n* (%)	
NBNC HBV HCV HBV + HCV	9 (7.5%) 77 (64.2%) 26 (21.7%) 8 (6.7%)
Metastasis ^a^, *n* (%)	
No Yes	109 (90.8%) 10 (8.3%)
Follow-up duration (months)	
Median, IQR	47.01 (15.08–108.68)

SD, standard deviation; IQR, interquartile range; Alcohol consumption, Yes-low (<30 gm/day for male, <20 gm/day for female) and Yes-high (>30 gm/day for male, >20 gm/day for female); AFP, alpha-fetoprotein; NBNC, non-B non-C; HBV, Hepatitis B virus; HCV, Hepatitis C virus; ^a^ *n* = 119 (refused to answer/unknown), ^b^ *n* = 118 (did not provide).

**Table 3:** Association of EpCAM and CD133 expression with clinicopathologic parameters.

Parameters (*n*)	EpCAM ^c^		*p* Value	CD133		*p* Value
	High-Expression *n*, (%)	Low Expression *n*, (%)		High-Expression *n*, (%)	Low Expression *n*, (%)	
Age (years)			**0.001**			0.367
<60 (65) ≥60 (55)	35 (55.6%) 13 (24.5%)	28 (44.4%) 40 (75.5%)		16 (24.6%) 9 (16.4%)	49 (75.4%) 46 (83.6%)	
Gender			0.106			0.155
Male (97) Female (23)	42 (45.2%) 6 (26.1%)	51 (54.8%) 17 (73.9%)		23 (23.7%) 2 (8.7%)	74 (76.3%) 21 (91.3%)	
Smoking, *n* (%) ^a^			>0.999			>0.999
No (59) Yes (60)	23 (41.1%) 24 (40.7%)	33 (58.9%) 35 (59.3%)		12 (20.3%) 13 (21.7%)	47 (79.7%) 47 (78.3%)	
Alcohol consumption, *n* (%) ^a^			0.148			0.904
No (70) Yes-low (18) Yes-high (31)	32 (47.8%) 7 (38.9%) 8 (26.7%)	35 (52.2%) 11 (61.1%) 22 (73.3%)		15 (21.4%) 3 (16.7%) 7 (22.6%)	55 (78.6%) 15 (83.3%) 24 (77.4%)	
AFP (ng/mL) ^b^			**0.007**			**0.043**
<400 (86) ≥400 (32)	28 (34.1%) 20 (65.5%)	54 (65.9%) 12 (37.5%)		14 (16.3%) 11 (34.4%)	72 (83.7%) 21 (65.6%)	
Tumor size (cm)			0.129			0.172
<5 cm (69) ≥5 cm (51)	24 (35.3%) 24 (50.0%)	44 (64.7%) 24 (50.0%)		11 (15.9%) 14 (27.5%)	58 (84.1%) 37 (72.5%)	
ES grade			0.330			0.819
Well (I and II) (79) Poor (III and IV) (41)	34 (44.7%) 14 (35/0%)	42 (55.3%) 26 (65.0%)		17 (21.5%) 8 (19.5%)	62 (78.5%) 33 (80.5%)	
Number of tumors			**0.036**			**0.013**
Solitary (70) Multiple (50)	22 (32.8%) 26 (53.1%)	45 (67.2%) 23 (46.9%)		9 (12.9%) 16 (32.0%)	61 (87.1%) 34 (68.0%)	
Vascular invasion			0.449			**0.039**
Absent (46) Vascular invasion (74)	17 (37.0%) 31 (44.3%)	29 (63.0%) 39 (55.7%)		5 (10.9%) 20 (27.0%)	41 (89.1%) 54 (73.0%)	
Pathology stage			0.302			0.076
Early (I) (33) Late (II, III and IV) (87)	11 (33.3%) 37 (44.6%)	22 (66.7%) 46 (55.4%)		3 (9.1%) 22 (25.3%)	30 (90.9%) 65 (74.7%)	
Cirrhosis			0.091			**0.013**
No (54) Yes (66)	27 (50.0%) 21 (33.9%)	27 (50.0%) 41 (66.1%)		17 (31.5%) 8 (12.1%)	37 (68.5%) 58 (87.9%)	
Viral status			**0.015** ^d^			0.590
NBNC (9) HBV (77) HCV (26) HBV + HCV (8)	1 (11.1%) 38 (51.4%) 6 (23.1%) 3 (42.9%)	8 (88.9%) 36 (48.6%) 20 (76.9%) 4 (57.1%)		2 (22.2%) 17 (22.1%) 6 (23.1%) 0 (0.0%)	7 (77.8%) 60 (77.9%) 20 (76.9%) 8 (100%)	
Metastasis ^a^			0.313			0.215
No (109) Yes (10)	41 (39.0%) 6 (60.0%)	64 (61.0%) 4 (40.0%)		21 (19.3%) 4 (40.0%)	88 (80.7%) 6 (60.0%)	

Alcohol consumption, Yes-low (<30 gm/day for male, <20 gm/day for female) and Yes-high (>30 gm/day for male, >20 gm/day for female); AFP, alpha-fetoprotein; ES grade, Edmondson–Steiner grade; NBNC, non-B non-C; HBV, Hepatitis B virus; HCV, Hepatitis C virus; ^a^ *n* = 119 (refused to answer/unknown); ^b^ *n* = 118 (did not provide); ^c^ *n* = 116 (expression undetermined); ^d^ Fisher exact test. Values in bold are statistically significant.

**Table 4:** Association of expression of CSC markers EpCAM and CD133, NRF2 and NRF2 target genes in TAE/TACE-treated HCC.

	EpCAM ^b^		*p* Value	CD133		*p* Value
	Low (68)	High (48)		Low (95)	High (25)	
NRF2 (*n*, %)			0.294			0.126
Low High	52 (76.5) 16 (23.5)	32 (66.7) 16 (33.3)		73 (76.8) 22 (23.2)	15 (60.0) 10 (40.0)	
NQO1 (*n*, %) ^a^			**0.011**			**0.047**
Low High	25 (36.8) 43 (63.2)	7 (14.6) 41 (85.4)		31 (33.0) 63 (67.0)	3 (12.0) 22 (88.0)	
HO-1 (*n*, %)			0.445			0.057
Low High	59 (86.8) 9 (13.2)	39 (81.2) 9 (18.8)		84 (88.4) 11 (11.6)	18 (72.0) 7 (28.0)	
GCLC (*n*, %)			0.330			0.636
Low High	42 (61.8) 26 (38.2)	34 (70.8) 14 (29.2)		62 (65.3) 33 (34.7)	18 (72.0) 7 (28.0)	
GCLM (*n*, %)			0.344			>0.999
Low High	43 (63.2) 25 (36.8)	26 (54.2) 22 (45.8)		55 (57.9) 40 (42.1)	15 (60.0) 10 (40.0)	

^a^*n* = 119 and ^b^ *n* = 116 (expression undetermined). Values in bold are statistically significant.

**Table 5:** Univariate and multivariate analysis for prognostic factors in post-TAE/TACE HCC patients.

Parameter	Overall Survival			
	Univariate Analysis		Multivariate Analysis	
	HR (95% CI)	*p* Value	HR (95% CI)	*p* Value
Gender (male/female)	1.248 (0.714–2.183)	0.437		
Age (≥60/<60)	0.989 (0.643–1.520)	0.958		
Smoking (Yes/No) ^a^	0.986 (0.641–1.516)	0.947		
Alcohol consumption ^a^	1.075 (0.694–1.666)	0.745		
No Yes-low Yes-high	1.00 (Ref) 1.206 (0.654–2.224) 1.008 (0.604–1.681)	0.549 0.976		
AFP (ng/mL) (≥400/<400) ^b^	1.829 (1.147–2.917)	**0.011**	1.168 (0.701–1.946)	0.552
Tumor size (≥5 cm/5 cm)	1.875 (1.221–2.880)	**0.004**	1.419 (0.840–2.399)	0.191
ES grade (Poor/Well)	1.412 (0.897–2.223)	0.136		
Number of tumor (Multiple/Solitary)	1.537 (0.998–2.369)	0.051		
Vascular invasion (Yes/No)	2.161 (1.363–3.426)	**0.001**	1.821 (1.124–2.9.52)	**0.015**
Pathology stage (Late/Early)	2.018 (1.208–3.370)	**0.007**	0.965 (0.414–2.245)	0.934
Cirrhosis (Yes/No)	0.823 (0.536–1.264)	0.373		
Viral status				
NBNC HBV HCV HBV + HCV	1.00 (Ref) 0.975 (0.419–2.268) 0.943 (0.376–2.363) 1.692 (0.545–5.256)	0.952 0.901 0.363		
Metastasis (Yes/No) ^a^	2.229 (1.112–4.470)	**0.024**	2.033 (1.002–4.125)	**0.049**
NRF2 (high/low)	0.695 (0.433–1.117)	0.133		
NQO1 (high/low) ^a^	1.126 (0.674–1.879)	0.651		
HO-1 (high/low)	0.788 (0.456–1.360)	0.391		
GCLC (high/low)	0.832 (0.537–1.290)	0.412		
GCLM (high/low)	0.664 (0.428–1.030)	0.068		
EpCAM (high/low) ^c^	1.108 (0.711–1.726)	0.652		
CD133 (high/low)	2.234 (1.394–3.579)	**0.001**	2.013 (1.223–3.314)	**0.006**

Alcohol consumption, Yes-low (<30 gm/day for male, <20 gm/day for female) and Yes-high (>30 gm/day for male, >20 gm/day for female); AFP, alpha-fetoprotein; ES grade, Edmondson–Steiner grade (Well, I and II; Poor, III and IV); Pathology stage early, I; late, II, III and IV; NBNC, non-B non-C; HBV, Hepatitis B virus; HCV, Hepatitis C virus; ^a^ *n* = 119 (refused to answer/unknown/undetermined), ^b^ *n* = 118 (did not provide), ^c^ *n* = 116 (undetermined). Values in bold are statistically significant.

## Data Availability

Not applicable.

## Supplementary Materials

The following supporting information can be downloaded at: https://www.mdpi.com/article/10.3390/medicina58020212/s1, Table S1: Gene expression patterns in TAE/TACE-treated HCC patients. Table S2: Association of NRF2 and its target genes with clinicopathological characteristics in TAE/TACE-treated HCC patients. Table S3: Combination of clinicopathological parameters with CD133 overexpression.
