# Supplementary material for: The Expression and Prognostic Value of Cancer Stem Cell Markers, NRF2, and Its Target Genes in TAE/TACE-Treated Hepatocellular Carcinoma
Source: Medicina (Kaunas). 2022 Feb 1;58(2):212. doi: 10.3390/medicina58020212 (PMC8879934; doi:10.3390/medicina58020212)
Supplement: Supplementary file 1 [file medicina-58-00212-s001.zip › medicina-1503641-supplementary.pdf]

SUPPLEMENTARY MATERIALS

Supplementary Table S1. Gene expression patterns in TAE/TACE-treated HCC patients

Genes	Patient *n*, (%)<br>*n* = 120
NRF2	
Low	**88** (73.3%)
High	**32** (26.7%)
NQO1 [a]	
Low	**34** (28.3%)
High	**85** (70.8%)
HO-1	
Low	**102** (85.0%)
High	**18** (15.0%)
GCLC	
Low	**80** (66.7%)
High	**40** (33.3%)
GCLM	
Low	**70** (58.3%)
High	**50** (41.7%)
EpCAM [b]	
Low	**68** (56.7%)
High	**48** (40.0%)
CD133	
Low	**95** (79.2%)
High	**25** (20.8%)

[a] *n* = 119 and [b] *n* = 116 (expression undetermined).

Supplementary Table S2. Association of NRF2 and its target genes with clinicopathological characteristics in TAE/TACE-treated HCC patients.

Parameters (*n*)	NRF2		***p***	NQO1 [a]		***p***	HO-1		***p***	GCLC		***p***	GCLM		***p***
	high	low	**value**	high	low	**value**	high	low	**value**	high	low	**value**	high	low	**value**
Age (years)			0.305			0.104			0.073			0.563			0.578
<60 (65)	20	45		50	14		6	59		20	45		29	36	
≥60 (55)	12	43		35	20		12	43		20	35		21	34	
Gender			0.612			0.608			0.520			0.470			0.249
Female (23)	5	18		15	8		2	21		6	17		7	16	
Male (97)	27	70		70	26		16	81		34	63		43	54	
Smoking [a]			0.677			0.684			0.799			0.051			0.458
No (59)	14	45		43	15		8	51		14	45		22	37	
Yes (60)	17	43		42	18		10	50		25	35		27	33	
Alcohol consumption [a]			0.876			0.845			>0.999			0.537			0.809
No (70)	18	52		51	18		11	59		20	50		30	40	
Yes-low (18)	4	14		12	6		2	16		7	11		6	12	
Yes-high (31)	9	22		22	9		5	26		12	19		13	18	
AFP (ng/mL) [b]			0.493			0.491			0.776			0.281			0.093
<400 (86)	25	61		60	25		14	72		31	55		40	46	
≥400 (32)	7	25		25	7		4	28		8	24		9	23	
Tumor size (cm)			>0.999			0.682			0.801			>0.999			0.852
<5 cm (69)	18	51		50	18		11	58		23	46		28	41	
≥5 cm (51)	14	37		35	16		7	44		17	34		22	29	
ES grade			0.390			>0.999			0.419			0.545			0.701
Well (I and II) (79)	19	60		56	22		10	69		28	51		34	45	
Poor (III and IV) (41)	13	28		29	12		8	33		12	29		16	25	
Number of tumors			>0.999			0.219			>0.999			0.560			0.852
Solitary (70)	19	51		46	23		10	60		25	45		30	40	
Multiple (50)	13	37		39	11		8	42		15	35		20	30	
Vascular invasion			0.291			0.145			>0.999			>0.999			0.849
Absent (46)	15	31		29	17		7	39		15	31		20	26	
Vascular invasion (74)	17	57		56	17		11	63		25	49		30	44	
Pathology stage			0.167			0.823			0.573			>0.999			0.837
Early (I) (33)	12	21		23	10		6	27		11	22		13	20	
Late (II, III and IV) (87)	20	67		62	24		12	75		29	58		37	50	
Cirrhosis			0.539			0.221			0.617			0.846			>0.999
No (54)	16	38		42	12		7	47		17	37		23	31	
Yes (66)	16	50		43	22		11	55		23	43		27	39	
Viral status			0.339			0.192			0.356			0.455			0.533
NBNC (9)	4	5		6	3		1	8		5	4		4	5	
HBV (77)	17	60		55	21		9	68		25	52		29	48	
HCV (26)	8	18		16	10		6	20		7	19		12	14	
HBV+HCV (8)	3	5		8	0		2	6		3	5		5	3	
Metastasis [a]			0.455			0.277			0.709			>0.999			0.741
No (109)	28	81		75	33		17	92		37	72		45	64	
Yes (10)	4	6		9	1		1	9		3	7		5	5	

Alcohol consumption, Yes-low (<30gm/day for male, <20 gm/day for female) and Yes-high (>30gm/day for male, >20 gm/day for female); AFP, alpha-fetoprotein; ES grade, Edmonson-Steiner grade; NBNC, non-B non-C; HBV, Hepatitis B virus; HCV, Hepatitis C virus;
[a]*n* = 119 (refused to answer/unknown/undetermined), [b]*n* = 118 (did not provide)

Supplementary Table S3. Combination of clinicopathological parameters with CD133 overexpression

Parameters	HR (95% CI)	*p* value	No. of patients
AFP/CD133 (≥400ng/ml/+ *vs* <400 ng/ml /-)	3.488 (1.777-6.847)	**<0.001**	11:72
Tumor size/CD133 (≥5cm/+ *vs* <5cm/-)	4.407 (2.320-8.371)	**<0.001**	14:58
Vas.inv/CD133 (vas.inv/+ *vs* no vas.inv/-)	3.915 (2.094-7.319)	**<0.001**	20:41
Path.stage/CD133 (late/+ *vs* early/-)	3.981 (2.051-7.727)	**<0.001**	22:30

AFP, alpha-fetoprotein; Vas.inv, vascular invasion; Path.stage, pathology stage (early, I; late, II, III and IV); +, CD133 high expression; -, CD133 low expression; HR, hazard ratio; CI, confidence interval; Values in bold are statistically significant.
